# Association between the choice of anesthesia method and reactivity to chemotherapy in children with acute lymphocytic leukemia: a preliminary retrospective cohort study

**DOI:** 10.1186/s40981-019-0283-5

**Published:** 2019-10-07

**Authors:** Takahiko Kaneko, Tetsuro Kagawa, Eri Ueshima, Munetaka Hirose

**Affiliations:** 1 Department of Anesthesiology and Pain Medicine, Hyogo College of Medicine, Mukogawa-cho 1-1, Nishinomiya, 663-8501 Japan; 2 Department of Anesthesiology, Kobe Children’s Hospital, Minatojima-Minamimachi 1-6-7, Chuo-ku, Kobe, 650-0047 Japan

## To the Editor,

Immune cell suppression by inhalation anesthetics can decrease patients’ response to chemotherapy for leukemia, although only a few reports have examined this [1]. We investigated the relationship between general anesthesia methods (propofol and sevoflurane) for tunneled central venous catheter (tCVC) insertion in pediatric patients with precursor B cell acute lymphocytic leukemia (BCP-ALL) and the residual leukemic blast cell count in peripheral blood on day 8 following prednisolone chemotherapy (day-8 PB blast cell count), an indicator of response to treatment [2].

The subjects of this single-center retrospective cohort study were BCP-ALL patients aged less than 18 years who underwent tCVC insertion under general anesthesia. During the research period of January 2012 through October 2018, the same chemotherapy protocol was used as the primary treatment for pediatric BCP-ALL at our institution. The exclusion criteria decided beforehand were discontinuation of prednisolone chemotherapy, age less than 1 year and/or positive Philadelphia chromosome cases requiring different treatment methods [2], and tCVC insertion after starting chemotherapy. The relationship between anesthesia method and the residual day-8 PB blast cell count was examined using logistic regression analysis and inverse probability of treatment weighting (IPTW) by propensity scoring.

A cohort of 57 patients with pediatric BCP-ALL was evaluated. The residual day-8 PB blast count was significantly lower in cases anesthetized with sevoflurane than propofol (crude odds ratio [OR] = 0.27; 95% confidence interval [CI], 0.085–0.86; *P* = 0.045), but no difference was observed when the values were adjusted for age (adjusted OR = 2.32; 95% CI, 0.70–7.65; *P* = 0.167) (Table 1). Furthermore, after adjustment by the IPTW method, there was no significant difference between the two groups (adjusted OR = 0.61; 95% CI, 0.16–2.3; *P* = 0.47) (Table 2).

**Table 1:** Characteristics of patients in the study cohort that were assessed using univariate and multivariate analysis as risk factors for the presence of residual blast cells in peripheral blood on day 8 following prednisone chemotherapy

			Univariate analysis		Multivariate analysis	
	No blasts (*n* = 24)	Residual blasts (*n* = 33)	Crude OR (95% CI)	*P* value	Adjusted OR (95% CI)	*P* value
Age (months)	53 (37–71)	74 (50–148)	1.01 (1.00–1.02)	0.074	1.008 (0.996–1.019)	0.18
Height (cm)	99.2 (91.2–110)	125 (103–149)	1.04 (1.01–1.07)	0.013		
Weight (kg)	16.3 (12.9–20.2)	19.8 (16–35)	1.05 (1.00–1.10)	0.053		
Gender (male) (%)	9 (37.5)	20 (60.6)	0.45 (0.15–1.33)	0.67		
ASA-PS≧III (%)	2 (8.3)	2 (6.1)	0.66 (0.086–5.02)	0.53		
Operation time (min)	29 (23–47)	27 (22–30)	0.97 (0.93–1.01)	0.15		
Anesthesia time (min)	83 (73–92)	83 (74–92)	0.99 (0.97–1.01)	0.45		
Anesthesia method (sevoflurane) (%)	12 (50.0)	7 (21.2)	0.27 (0.085–0.86)	0.045	2.31 (0.70–7.65)	0.17
WBC > 50,000/μL at diagnosis (%)	2 (8.3)	8 (24.2)	3.23 (0.619–16.9)	0.18		
Central nervous invasion (%)	1 (4.2)	5 (15.2)	3.79 (0.413–34.8)	0.39		
Mediastinal invasion (%)	0 (0)	0 (0)				
Hypodiploid (%)	1 (4.2)	0 (0)	0.393 (0.284–0.554)	0.40		
Genetic risk (%)	1 (4.2)	6 (18.2)	4.714 (0.528–42.10)	0.22		

Data are expressed as median (25th–75th percentile) or *n* (%). Residual blast cells: leukemic blast cell count in peripheral blood on day 8 following prednisone chemotherapy > 0/μL. Hypodiploid: fewer than 45 chromosomes. Genetic risk: Evidence of MLL-AF4, MLL reconstitution and/or E2A-HLF

*OR* odds ratio, *CI* confidence interval, *ASA-PS* American Society of Anesthesiologists physical status, *WBC* white blood cell count

**Table 2:** Relationship between the anesthesia method and the presence of residual blast cells in peripheral blood by inverse probability of treatment weighting (IPTW) using propensity score

	Before weighting			After weighting		
	Group S (*n* = 19)	Group P (*n* = 38)	Crude OR (95% CI)	*P* value	Adjusted OR (95% CI)	*P* value
Residual blasts	7 (36.8)	26 (68.4)	0.27 (0.085–0.86)	0.045	0.61(0.16–2.3)	0.47
	Before weighting			After weighting		
	Group S	Group P	*P* value	Group S	Group P	*P* value
Blast count (/μL)	0 (0–30.0)	31.5 (0–349)	0.014	1.00 (0–72.0)	33.4 (0–314)	0.20

Data are expressed as *n* (%) or median (25th–75th percentile). Group S patients received general anesthesia with sevoflurane and group P received propofol. Residual blasts: number of patients in whom leukemic blast counts in peripheral blood on day 8 following prednisone chemotherapy were > 0/μL. Blast count: leukemic blast cell count in peripheral blood on day 8 following prednisone chemotherapy

*OR* odds ratio, *CI* confidence interval

In this study, the number of patients with residual leukemic blast cells was significantly lower in patients receiving sevoflurane than in those receiving propofol. The reason for this result contrary to the hypothesis may be that the children in the propofol group were significantly older. Therefore, the general anesthetics were not associated with the presence and count of residual day-8 PB blast cells after adjustment. One of the possible reasons for the lack of a relationship between anesthetics and the presence of residual day-8 PB blast cells is the short duration of anesthesia (approximately 80 min). Another reason is that many patients classified as the propofol group received sevoflurane at the time of procedures such as bone marrow aspiration and lumbar puncture. Use of thiopental, which has an inhibitory effect on immune cells [3], in most cases for induction of general anesthesia, might have affected the results.

This research has many limitations. First, it was a retrospective study with a small number of cases, and it may have lacked statistical power or involved confounding factors that could not be adjusted. Second, although recurrence and survival rates are considered more important outcomes, alternative endpoints were used in this study.

The results of the present study have demonstrated no superiority of sevoflurane and propofol.

## Data Availability

The datasets used and/or analyzed during the current study are available from the corresponding author on reasonable request.

## Abbreviations

BCP-ALL: Precursor B cell acute lymphocytic leukemia
CI: Confidence interval
IPTW: Inverse probability of treatment weighting
OR: Odds ratio
PB: Peripheral blood
tCVC: Tunneled central venous catheter

## References

[1] Di N, Guo Y, Ding N (2019). Effect of combined propofol-sevoflurane anesthesia on immune function in pediatric patients with acute lymphoblastic leukemia. Oncol Lett..

[2] Möricke A, Reiter A, Zimmermann M, Gadner H, Stanulla M, Dördelmann M, Löning L, Beier R, Ludwig W-D, Ratei R, Harbott J, Boos J, Mann G, Niggli F, Feldges A, Henze G, Welte K, Beck J-D, Klingebiel T, Niemeyer C, Zintl F, Bode U, Urban C, Wehinger H, Niethammer D, Riehm H, Schrappe M, German-Austrian-Swiss ALL-BFM Study Group (2008). Risk-adjusted therapy of acute lymphoblastic leukemia can decrease treatment burden and improve survival: treatment results of 2169 unselected pediatric and adolescent patients enrolled in the trial ALL-BFM 95. Blood..

[3] Melamed R, Bar-Yosef S, Shakhar G, Shakhar K, Ben-Eliyahu S (2003). Suppression of natural killer cell activity and promotion of tumor metastasis by ketamine, thiopental, and halothane, but not by propofol: mediating mechanisms and prophylactic measures. Anesth Analg..

